# Effect of Storage Temperature on Sliced Vacuum-Packed Dry-Cured Portuguese Sausage (*Painho de Porco Preto*)

**DOI:** 10.3390/foods15071119

**Published:** 2026-03-24

**Authors:** Sofia Trindade, Ana Cristina Agulheiro-Santos, Alberto Ortiz, Lucía León, Maria Freire, David Tejerina, Miguel Elias

**Affiliations:** 1 MED Mediterranean Institute for Agriculture, Environment and Development & CHANGE Global Change and Sustainability Institute, Institute for Advanced Studies and Research, Universidade de Évora, 7004-516 Évora, Portugal; 2 MED Mediterranean Institute for Agriculture, Environment and Development & CHANGE Global Change and Sustainability Institute, Departamento de Fitotecnia, Escola de Ciências e Tecnologia, Universidade de Évora, 7004-516 Évora, Portugal; 3 Meat Quality Area, Centre of Scientific and Technological Research of Extremadura (CICYTEX-La Orden), Junta de Extremadura, Ctra, A-V, Km372, 06187 Guadajira, Spain; alberto.ortiz@juntaex.es (A.O.);

**Keywords:** iberian sausage, fatty acids profile, storage temperature, characterization

## Abstract

*Painho de Porco Preto* is a traditional product of the Alentejo region, made with cuts of Alentejano autochthonous breed pigs. The objective of this study was to evaluate how different storage temperatures (4 °C and room temperature (20 ± 2 °C)) could influence the quality and safety of the sliced vacuum-packed *Painho de Porco Preto*, throughout 6 months of storage. Analyses included physicochemical parameters, microbiological, and sensory analysis. Throughout storage, the product showed low TBARS values (<3 MDA/kg) and stable tocopherol levels under both storage conditions, although the samples at room temperature performed slightly better. a_w_ and pH values were higher for samples stored at 4 °C, which influenced the results of some parameters. Color coordinate b* had an increase in values by the end of storage for the fat portion of the slices, but the rest of the parameters stayed stable. Nitrate/nitrite contents remained within expected ranges for dry-cured sausages. Microbiological analyses confirmed the absence of major pathogens during the study period, while variations in growth were observed depending on storage temperature. In sum, the results indicate that sliced vacuum-packaged *Painho de Porco Preto* can maintain acceptable quality and safety for 6 months at room temperature. These findings provide useful information for the meat industry by supporting the optimization of storage strategies and shelf-life management for sliced traditional dry-cured sausages.

## 1. Introduction

The necessity to preserve food, particularly meat, for extended periods of time led to the development, over centuries, of preservation methods that ensured good nutritional and sensory characteristics and safety in consumption. In Mediterranean countries with this objective in mind, production and consumption of dry-cured sausages are traditional and highly appreciated. Some of these traditional and regional products play an important role in the country’s cultural aspects, economy, and food supply [1]. 

Fermentation has long been used as a traditional preservation technique, allowing for stable meat products and preserving different and typical sensorial profiles of the products. This differentiation is important from the point of view of promoting regional products, giving them desirable authenticity. So spontaneous fermentation is often used in traditional products obtained in small-scale plants [2]. Another complementary preservation technique applied to this meat product is dry curing—salting of muscle tissue to promote water loss through osmosis and controlled air-drying. This reduces water activity (a_w_), promotes microbial growth control, and contributes to good flavor and adequate texture. The reduction in water activity and the action of salting ensure microbiological safety and thus a desirable increase in shelf life, maintaining their sensory properties, allowing these products to be consumed safely and pleasurably [3].

The shelf life of these products is typically determined by complex interactions between intrinsic factors (a_w_, pH, salt content, and fat composition) and extrinsic factors, such as interactions with competing background microbiota and gas atmosphere, with storage temperature being one of the most critical variables [4,5,6]. Refrigeration at approximately 2 to 5 °C has been extensively documented as an effective strategy for slowing microbial activity and reducing spoilage in vacuum-packaged cured meats, while room temperature (20 ± 2 °C) storage increases the risk of microbial growth and accelerates lipid oxidation, compromising both product safety and sensory quality [7,8,9,10]. 

In studies conducted with vacuum-packed Poličan dry fermented sausage during 120 days of storage at two different temperature conditions, 5 and 15 °C, no differences were observed in sensory evaluation and in physical/chemical and microbiological parameters [11]. The results obtained in a study of Sardinian fermented sausage by Siddi et al. [6] relating to the physical-chemical, microbiological, and sensory characteristics of the product did not reveal any specific problems during the 120 days at refrigeration temperature (4 °C ± 1 °C) after packaging, preserving product quality as demonstrated by the typical physicochemical parameters such as pH < 5.5, a_w_ ≤ 0.920, sodium chloride, nitrates, and nitrites.

A very specific and innovative study conducted by Majcherczyk et al. [12] of sausage snacks produced from traditional homemade sausage by air-drying reported the reduction in a_w_, combined with the modified oxygen-free atmosphere packaging used in this study, allowed the products to maintain their high quality for at least 70 days when stored at 4 °C, and 4 weeks at room temperature (20 °C), which, according to the authors, is due to the nature of the raw material, which contains natural antioxidants in the spices and has been cured and smoked. 

Ščetar et al. [13] tested the shelf life of sliced dry fermented sausage during storage at different temperatures (4, 22, and 37 °C), in two types of packaging: vacuum and nitrogen (100% N_2_) during 120 days of storage. Packaging materials were analyzed for oxygen permeability. Sensory quality limited the shelf life of the studied product before the limiting effects of microbial proliferation occurred. While packaging type and method (vacuum/N_2_) showed no significant effect on pH and a_w_, both temperature and storage days significantly influenced these parameters. Sensory evaluation showed sensitivity to different packaging materials, storage time, and temperature. The results obtained in sensory evaluation indicated that the shelf life of sausages stored at 4 °C in vacuum packaging was 95 days; at 22 °C, it was reduced to 30 days. At 37 °C, regardless of packaging material and method, all samples were found to have a shelf life of 15 days.

As a preservation strategy, vacuum packaging serves to enhance stability and extend the shelf life of meat products. The environment created in the meat by vacuum packaging, with low oxygen content, the presence of NaCl and NaNO_2_, and reduced a_w_, helps to inhibit the growth of Gram-negative bacteria that cause spoilage, such as *Pseudomonas*, *Acinetobacter*, and *Enterobacter*, and minimize inappropriate changes caused by lipid oxidation [14]. The presence of pathogens such as *Escherichia coli* O157:H7, *Staphylococcus aureus*, *Salmonella*, and especially *Listeria monocytogenes*, is a serious concern [15,16,17,18]. This last one is a psychrotrophic pathogen that can thrive and multiply at temperatures as low as 0 °C to 4 °C, usually used as storage temperatures [19].

In addition to safety and quality, food products must be aligned with consumer demand. These preferences influence how food is processed, preserved and presented to consumers, placing emphasis on shelf life. Consequently, stakeholders are continuously seeking innovative packaging and preservation alternatives. Specifically for dry-cured sausages, sliced packaging meets the need for convenience and boosts profitability in the ready-to-eat sector.

The object of this study is *Painho de Porco Preto*, a dry-cured sausage, traditional in the Alentejo region, south of Portugal. This product is characterized by being a mixture of minced meat seasoned with sea salt, garden paprika, and fresh garlic. According to the manufacturing company, the usual shelf life period of *Painho de Porco Preto* is 6 months, at a recommended storage temperature between 5 and 15 °C [20]. And while it is recommended to store these products at refrigeration temperatures to preserve quality and extend the shelf life, since higher temperatures could lead to a loss of quality, retail stores will often display and sell them at room temperature for marketing convenience, which might cause safety concerns involving some microorganisms [7,10].

Despite the existence of substantial research on temperature effects in cured meats, limited attention has been given to the conservation of sliced *Painho de Porco Preto*. 

Therefore, this study aims to investigate the physicochemical, microbiological, and sensory changes in sliced vacuum-packaged *Painho de Porco Preto* stored at 4 °C and at room temperature (20 °C), considering the possibility of extending the shelf life of sliced vacuum-packed product to 180 days (six months) of storage under reasonably predictable conditions.

The results of this study should contribute to improving the conservation practices of the traditional Portuguese product *Painho de Porco Preto*. By aligning its presentation with current consumer preferences, sliced and packaged, this research aims to support the industry and facilitate entry into new markets.

## 2. Materials and Methods

### 2.1. Painho Samples and Experimental Design

A total of 270 vacuum-packaged samples of *Painho de Porco Preto* dry-cured sausages (100 g format) were obtained from a manufacturing company, “Salsicharia Estremocense S.A.”, located in the Alentejo region, Portugal, and used in the present study. The samples were obtained from three different production batches to account for the potential variability associated with both the raw material and the industrial manufacturing process. *Painho* sausages were produced as follows: the initial batter was prepared using lean meat (70–80%) and back fat (20–30%) from Alentejano autochthonous breed pigs. After mechanical mincing, the mixture was seasoned with red pepper (*Capsicum annuum* L.) paste (6%), water (3%), garlic (*Allium sativum* L.) paste (1%), salt (0.7%), disodium diphosphate (0.03%), pentasodium triphosphate (0.03%), NaNO_3_ (0.03%), KNO_3_ (0.008%) and KNO_2_ (0.007%). The prepared mass was then kept under refrigeration (4 ± 2 °C) and high relative humidity (>85%) for 48 h to allow ripening. Subsequently, the sausage mixture was stuffed into artificial casings (50–60 mm diameter) and subjected to a smoking process for 12 days, with slow combustion of holm oak wood, at temperatures ranging from 16 to 27 °C. Finally, the sausages were transferred to an industrial chamber at a temperature of 15 to 17 °C and a relative humidity of 65 to 80% for 21 days. At the end of the technological process, the dry-cured sausages were sliced (2 mm thickness) and vacuum-packaged using a vacuum packaging machine (Ulma, Mod. FSD 701). The packaging material consisted of laminated film characterized by a 52 μm thickness; density: 1.04 kg/dm^3^; oxygen transmission rate of <3 cc/m^2^/day; and water vapor transmission rate of <15 g/m^2^/day.

Thereafter, a subset of 135 packages was stored under conventional refrigeration conditions (4 ± 2 °C), while another subset of 135 packages was kept at room temperature (20 ± 2 °C) for a period of 6 months. Analyses were performed on the first week of the study, from now on referred to as time 0, and after 2, 4, and 6 months of storage. The analysis done at time 0 (T0) served the purpose of characterizing the product and establishing a baseline to compare the evolution of the studied parameters. 

### 2.2. Initial Characterization

Dry matter (DM) and ash were measured following the AOAC method [21] and the chloride content (NaCl) was measured using the Volhard method [22]. The fat content was analyzed according to [23]. The protein content was determined from the nitrogen content (N × 6.25) using the Kjeldahl method [24] and results were expressed as g/100 g.

### 2.3. pH and a_w_

pH values were measured in duplicate in each homogenized sample with a pH meter (VWR pHebomenal, pH 1100L; Radnor, PA, USA). To determine water activity (a_w_), a Rotronic Hygrolab (Rotronic, Bassersdorf, Switzerland) was used, with an HC2-AW probe maintained at a temperature of 25 °C.

### 2.4. Instrumental Color

The color of the samples was analyzed using a Cr−400 Konica Minolta colorimeter, using the CIE L*a*b* color space (L*: lightness, a*: redness, b*: yellowness). Two measurements in the surface of the *Painho* were taken, one targeting the meat and the other the fat portion of the slice. Five slices of each package were analyzed. The data were processed with the software Spectra MagicTM NC version 10.0.

### 2.5. Antioxidant Content

Tocopherols were obtained by saponification using an 11.5% KOH solution prepared in EtOH/H_2_O (55:45), with ascorbic acid added to prevent oxidation. The extracts were then analyzed on an Agilent 1100 Series HPLC system (Agilent Technologies, Santa Clara, CA, USA) fitted with a Kromasil silica column (5 µm, 150 × 4.6 mm; Symta, Madrid, Spain) and a Kromasil Silica Guard column (10 µm; Symta, Madrid, Spain). The mobile phase consisted of hexane:isopropanol:ethanol (98.5:1:0.5, *v*/*v*) delivered at 1.0 mL/min, and fluorescence detection was set to 295 nm (excitation) and 330 nm (emission) (Agilent Technologies Series 1200). Peaks were identified and quantified by comparison with α- and γ-tocopherol calibration standards (0.2–14 µg/mL), and concentrations were reported as µg per gram of sample (µg/g).

### 2.6. Lipid and Protein Oxidation

Lipid oxidation was determined by the 2-thiobarbituric acid (TBA) assay following [25]. TBARS were obtained from a calibration curve prepared with 1,1,1,3-tetraethoxypropane (TEP) and reported as mean values of mg malondialdehyde (MDA) per kg of sample.

Protein oxidation was evaluated by quantifying carbonyl groups generated after derivatization with 2,4-dinitrophenylhydrazine (DNPH) in 2 N HCl, as described by Oliver et al. [26]. Carbonyl content was calculated from the amount of DNPH incorporated, using an extinction coefficient of 21.0 mM^−1^ cm^−1^ at 370 nm for protein hydrazones. Protein concentration was measured spectrophotometrically at 280 nm using bovine serum albumin (BSA) as the standard, and protein oxidation was finally reported as nmol carbonyls per mg protein.

### 2.7. Fatty Acid Profile

Lipids were first extracted following the method of Folch et al. [23]. Fatty acid methyl esters (FAME) were then produced by acid transesterification using methanolic sulfuric acid (5% H_2_SO_4_ in methanol) and sodium methoxide, as described by [27]. The FAMEs were analyzed by gas chromatography (Agilent 6890; Agilent Technologies, Santa Clara, CA, USA) equipped with a DB-23 capillary column (60 m × 0.25 mm i.d., 0.5 µm; Agilent Technologies), using a flame ionization detector (FID). Injector and detector temperatures were set at 260 °C and 280 °C, respectively. The oven was held at 185 °C for 2 min, ramped to 220 °C at 5 °C/min, and maintained at 220 °C for 40 min. Fatty acids were assigned by matching their retention times to those of a reference FAME mixture (Sigma-Aldrich, Supelco 37 Component FAME Mix, CRM47885; St. Louis, MO, USA), and were quantified on this basis. Results were reported as mg FAME per g of fat.

### 2.8. Nitrite and Nitrate Content

The nitrate content was determined using the brucine colorimetric method [28]. The sample was homogenized and subjected to a deproteinization treatment by sequentially adding sodium tetraborate, Carrez I, and Carrez II in order to precipitate proteins and obtain a clear extract suitable for analysis. After separating the precipitate by centrifugation, the extract was reacted with brucine in sulfuric acid, developing a yellow color proportional to the nitrate concentration. The absorbance was measured spectrophotometrically and quantified using a calibration curve prepared with nitrate standards, with the results expressed in mg/kg of sample.

The nitrite content was determined using the Zambelli colorimetric method, employing the same extract obtained after the deproteinization stage (sodium tetraborate + Carrez I + Carrez II). In the presence of the Zambelli reagent (a mixture of hydrochloric acid, sulfanilic acid, phenol, and ammonium chloride), the nitrites present react to form a yellow color, the intensity of which is proportional to the concentration of NO_2_^−^. The absorbance was determined by spectrophotometry, and the concentration was calculated by comparison with a calibration curve prepared with nitrite standards, expressing the results in mg/kg of sample.

### 2.9. Microbiological Analyses

The different microbiological parameters were analyzed following international standards and established procedures, and the results are expressed as log colony-forming units per gram of product (cfu)/g. Decimal dilution series were prepared in buffered peptone water, plated, and incubated as follows: mesophiles in Tryptone Glucose Extract (TGE) Agar at 30 °C for 48 h [29]; lactic acid bacteria (LAB) in de Man, Rogosa, and Sharpe (MRS) Agar at 30 °C for 48 h under anaerobic conditions in an AnaeroJar using an AnaeroGen sachet [30]; staphylococci in Mannitol Salt Agar (MSA) at 37 °C for 48 h; yeasts and molds in Rose Bengal Chloramphenicol Agar at 25 °C for 5 days [31]; Enterobacteriaceae in Violet Red Bile Glucose (VRBG) Agar at 30 °C for 48 h [32]; *Listeria* spp. in ALOA plates at 37 °C for 48 h [33]; and *Salmonella* spp. detection was performed with VIDAS (bioMérieux, Marcy-l’Étoile, France) [34].

### 2.10. Sensory Analysis

The tasting panel was composed of eight members who were trained in accordance with ISO 8586-1:2001 [35]. The panelists evaluated a maximum of six samples per session, which consisted of slices randomly distributed in white dishes, each identified with a random three-digit number. Panelists evaluated the different parameters, usually referred to as descriptors, by means of a quantitative descriptive analysis. The 12 descriptors evaluated were: color intensity, off-colors, marbled appearance, aroma intensity, off-aromas, hardness, fibrosity, succulence, flavor intensity, off-flavors, salt perception, and this list of descriptors is complemented by an overall assessment. The panelists rated the descriptors in a scale ranging from 0 (“not perceived”) to 100 (“maximum perception”). Crackers and mineral water were supplied to the panelists as palate cleansers.

### 2.11. Statistical Analysis

Statistical treatment was performed using Statistica software version 13.0 (StatSoft, Inc., Dell, Tulsa, OK, USA). Analysis of variance (ANOVA) was performed for a significance level of 0.05. The means were compared and the differences between groups were identified based on Tukey’s honestly significant difference (HSD) test (*p* < 0.05). Tables were prepared for each parameter evaluated, with the results obtained in the ANOVA and Tukey’s multiple comparisons test, using letters of the alphabet to indicate the significant differences found.

## 3. Results and Discussion

### 3.1. Initial Characterization

*Painho* sausages were characterized in T0, and Table 1 summarizes the results obtained. The *Painho* has a low a_w_, which is typical of dry-cured sausages and a good indicator of microbiological stability [1]. The values of moisture, and chlorides are similar to the ones found in the other study of dry-cured Iberian sausages [36]. In their study, Elias and Carrascosa [23] observed values ranging between 27.2 and 59.3% of moisture; and values of 0.2% to 6% of chlorides. The protein values were, however, higher than the value of 19.2 g/100 g found in Marcos et al. [5], but slightly lower than the ones observed in the study of “Salchichón” sausages [22]. The total fat value is slightly above the average value of 26.8 g/100 g that Marcos et al. [3] obtained in their study, but it is very similar to the values of 27.30 to 29.72% found in dry-cured Iberian sausages [22].

### 3.2. Color

In Table 2, it is possible to observe that the only color coordinate that showed any significant difference was b* measured in the fat portion of the slices. The samples stored at room temperature exhibited a decrease in b* until month 6 where an increase was observed. The samples stored at 4 °C appeared to have the opposite behavior. The increase in the values of the color parameter b* indicates the appearance of a yellow tone, while the decrease signifies a color shift to blue. No significant differences were found for the other color coordinates. Overall, the color of the *Painho* slices remained stable during this trail, which was surprising since usually slicing should decrease the color stability of dry-cured meat products because the surface exposed to light or oxygen is increased [10]. This stability, could be attributed to the vacuum packing, since it reduces the oxygen permeability, leading to reduced oxidations [37], and it also allows for an equalization of moisture inside the packaging meaning that there is less drying of the slices [12]. These factors are important, because increases in yellowness, such as the one observed in the color parameter b*, are associated with a higher lipid oxidation and rancidness perception [38]. The reduced oxidation factor also favors the stability of values of the color parameter a*. This parameter is related to the content of myoglobin that is present in the samples, and the values usually decrease when there is a rise in the levels of metmyoglobin, which happens when oxidation transforms deoxymyoglobin into metmyoglobin [39]. The further drying of the samples can cause changes in the L* value due to the loss of water [12,39], which despite not showing significant differences does showcase higher values for the meat portion of the samples stored at 4 °C, indicating that some water loss could have occurred in the room temperature samples resulting in lower values of L*. 

The behavior observed is similar to the one described by Cava et al. [8] in their study; however, the L*, a*, and b* values obtained in their study are overall lower and they did not find any significant differences for any of them. The study published by Trejo et al. [10] exhibits similar values in the L* color coordinate when compared to those observed in the meat portion of the slices, the other a* and b* values are, however, lower than those of the present study. Nevertheless, Trejo et al. [10] also observed color stability in their sliced dry-cured sausages. In the opinion of the authors that color stability is caused by the addition of garlic during the sausage production, since garlic is rich in antioxidant compounds [40,41] which could stabilize color changes during the storage. 

### 3.3. pH and a_w_

Table 3 summarizes the results for pH and a_w_. pH values play an important role in limiting the growth of microorganisms, since most microorganisms require a neutral pH for their development [42]. pH mean values showed significant differences throughout storage time between the two storage temperatures, with the values of pH from the sausages stored at room temperature being lower than those observed in the samples stored at 4 °C. Overall, the *Painho* samples showed values of pH between 5.6 and 6.2, results which were similar to those published in other studies [4,43,44]. Although the values remained mostly stable through the storage time, there are some variations which could be caused by the production of organic acid by lactic acid bacteria (LAB), leading to a slight decrease in pH; while an increase in pH values could be caused by the liberation of metabolites resulting from bacterial activity [12,45,46]. 

Regarding a_w_, significant differences were observed throughout storage time between the two storage temperatures. Values were slightly higher for the samples stored at 4 °C, with the highest value observed being 0.86 ± 0.0, while values were lower for the samples stored at room temperature. This result was expected since the higher storage temperature can cause loss of water in the samples, and consequently, the a_w_ will be lower.

It is important to analyze these values, since a_w_ measures the quantity of water available to microbial metabolisms [47]. The lower the amount of water available, the lower the possibility of microbial growth, so it is essential for good stability that a_w_ values remain low. Some studies claim that the preservation of dry meat products, such as *Painho* sausages, relies on having an a_w_ below 0.91, since growth and production of toxins is not possible by the pathogens that raise concerns in the food industry [47,48]. It is known that each microorganism has its own ideal growth condition, and therefore, they have different a_w_ values that they can withstand. Pathogens such as *Staphylococcus aureus* can still grow in situations where the a_w_ is at a value of 0.86 [49], while yeasts and molds can still grow when a_w_ reaches values of 0.70 to 0.62 [50,51]. However, while the a_w_ plays an important role in limiting the growth of pathogens, it is still necessary to control other factors such as temperature and pH, since those also influence the ability of these microorganisms to develop [47,49].

### 3.4. Antioxidant Content

Tocopherols are primary forms of vitamin E with antioxidant capacity, preventing lipid oxidation and consequently rancidity [52,53]. According to Seppanen et al. [54] α-tocopherol generally has better antioxidant activity than ɣ-tocopherol, but at higher concentrations, ɣ-tocopherol is a more active antioxidant. Table 4 shows the results of the sausages’ antioxidant content, and as can be observed, no significant differences were found. The α-tocopherol values ranged between 6.8 ± 0.5 and 7.3 ± 0.7 µg/g, while the values of ɣ-tocopherol remained very stable at 0.4 ± 0.1 µg/g, only lowering on one occasion to the value of 0.3 ± 0.0 µg/g.

The α-tocopherol values present in Table 4 are lower than those presented in some other studies [55], and overall may be considered low values [52] although above the value obtained by Alves et al. [56], Pateiro et al. [57], and Fernández-López et al. [58]. Higher α-tocopherol concentrations did not affect antioxidant capacity. As for the ɣ-tocopherol values, they were higher than the values observed by Magrinyà et al. [59]. Those authors also observed a decrease in tocopherols during storage, which can also be observed in this study, especially in the α-tocopherol values, with a bigger decrease happening in the samples stored at 4 °C. This behavior was also observed by Rey et al. [60], although in their study, γ-tocopherol was more susceptible to degradation, a difference that could be explained by the different characteristics of the raw materials used, since the feeding and rearing of the animals directly influence the content of tocopherols and how they decrease [60].

### 3.5. Lipid and Protein Oxidation

Lipids are known for containing essential fatty acids and fat-soluble vitamins, besides that, they also provide energy to the biological processes of the body; therefore, they represent compounds of great importance for suitable human nutrition [61]. They are present in all types of meat, and characteristics such as flavor, aroma profile, tenderness, and juiciness are influenced by lipids [62]. Therefore, the degradation of these compounds, known as lipid oxidation, affects those characteristics in the meat products, leading to the appearance of off-flavors, but also causing changes in color, texture, and nutritional value [63]. As for the protein oxidation, the results are not only a deterioration of the meat color and texture, but also the loss of essential amino acids and a decrease in the digestibility of proteins [64].

For lipid oxidation, the values ranged between 0.7 ± 0.1 and 1.1 ± 0.3 (mg MDA/kg sample) (Table 5), and no significant differences were found. There was an initial spike in the values of lipid oxidation, from T2 to T3, which, for the samples stored at 4 °C, matches the decrease in α-tocopherol values (Table 4), as tocopherols are known for having the capacity to prevent lipid oxidation. Overall, it is possible to observe that values of lipid oxidation were slightly higher in the samples stored at 4 °C, which were the same samples that showed a bigger decrease in α-tocopherol values. Lipid oxidation promotes rancidity problems, which are considered unpleasant for consumers [65], and while there is no standardized criterion [12], values below 2 mg MDA/kg, such as those obtained, are preferable since they indicate good product quality [66].

Regarding protein oxidation, significant differences were observed, with values increasing in samples stored at 4 °C. Oxidation values are akin to the values found in some other studies of dry-cured sausages [7,10,37]. The increase in protein oxidation at low temperatures contrasts with the findings of Cava et al. [8], who reported rising oxidation levels at both 4 °C and 18 °C, with a more pronounced trend observed at the higher storage temperature. On the other hand, Carrapiso et al. [7] observed an increase in protein oxidation in both storage temperatures, with higher values at 4 °C. The difference in results could be justified by the high hydrostatic pressure treatment that was done in the trial of Cava et al. [5], which causes higher values of protein oxidation [5]. In addition, differences in the manufacturing process, namely characteristics of raw materials and packaging, can affect the results, since lipid and protein oxidation depend on these factors [64].

Unfortunately, establishing relations between lipid and protein oxidation values and other factors is not, however, always possible because, in general, the TBARS values of meat tend to increase over the storage period, reaching a maximum value before beginning to decline. This curvilinear change over time, therefore, makes it difficult to draw conclusions about the cause of certain behaviors and observations [54,67].

### 3.6. Fatty Acid Profile

There were no significant differences in the fatty acid profile, apart from the Margaroleic acid (Table 6), which increased in the samples stored at room temperature. The major fatty acids in the *Painho* sausages were oleic (C18:1), palmitic (C16:0), and stearic (C18:0), followed by linoleic (C18:2) and palmitoleic (C16:1) acid. It is also possible to observe that the most abundant fatty acids were MUFA (monounsaturated fatty acids) and SFA (saturated fatty acids), followed by PUFA (polyunsaturated fatty acids). In general, the values for the fatty acids were higher in the samples stored at 4 °C, which was also observed in other studies [68,69].

It is also important to notice that high percentages of MUFA and PUFA cause products to become highly sensitive to oxidation [70]; therefore, they are some of the main factors responsible for the occurrence of lipid oxidation [61]. The results of the lipid oxidation (Table 5) showed a similar behavior of increasing and decreasing as did the values of MUFA and PUFA, with the variations occurring in a close timeframe for the most part. This could reflect the influence that these fatty acids have on the lipid oxidation of the sausages.

However, polyunsaturated fatty acids oxidize faster than monounsaturated fatty acids, meaning that the linoleic acid (C18:2) oxidation occurs faster than that of the oleic acid (C18:1), which, in turn, occurs slower than that of the oxidation of the linolenic acid (C18:3). The *Painho* samples have lower values of PUFA compared to the MUFA and higher values of C18:1 than C18:2 and C18:3; hence the oxidation of the fatty acids does not happen as fast as it would if the linoleic acid existed in more prevalence.

The prevalence of these acids was observed in other studies of dry-cured sausages [1,71,72], although the values observed in these studies are lower than those seen in our analysis. These differences are expected since differences in pig diet, breed and genetic lines, rearing system, and meat cut utilized in the production can affect the fatty acid composition [10,72]. Fatty acid composition is a main factor of lipid oxidation, alongside the content of fat, due to the fact that unsaturated fatty acids are easily oxidized [61].

### 3.7. Nitrite and Nitrate Content

Table 7 summarizes the results of the nitrate and nitrite levels of the *Painho* sausages. Significant differences were only found in the nitrite results. However, it is possible to observe that the nitrate values ranged from 43.8 ± 8.8 to 95.7 ± 5.9 mg/kg of product, while the nitrite values ranged from 0.2 ± 0.0 to 1.3 ± 0.6 mg/kg of product. Nitrite and nitrates are fundamental in dry-cured sausages, contributing not only to the development of the desired red color but also to the aroma formation, preventing rancidity, and exerting antimicrobial activity [73,74,75,76,77]. Despite their benefits, their usage and concentration in foods are restricted due to potential health implications [78]. Nitrite values are also usually lower due to the fact that nitrite can oxidize into nitrate [79].

While numerous studies have investigated the effects of reducing nitrates and nitrites in dry-cured sausages, there is a scarcity of research evaluating their content and evolution during storage, which complicates the comparison with our findings.

Still, the values of nitrate obtained seemed to be in line with the values observed in other studies of dry-cured products [74,80,81,82]. Their decrease was also previously observed [77,79]. More importantly, the values of nitrate observed are below 150 mg/kg, which is the maximum permitted by law [83]. It is also possible to observe that values tend to be higher in the samples stored at 4 °C, and the reason behind this could be the higher pH value found in refrigerated samples, alongside the higher temperature of the room-temperature samples. Higher pH values result in a slower disappearance of nitrite and nitrate, while high temperatures favor a loss of nitrite and a reduction in the formation of nitrate [79,84].

### 3.8. Microbiological Analyses

Table 8 shows significant differences in all microbiological parameters. No mold, enterobacteria, *Salmonella* spp., or *Listeria* spp. were detected in the samples. Staphylococci and yeast were the only parameters that showed slight increases through storage time in the samples stored at 4 °C. Overall, values are higher in the samples stored at 4 °C, something that was also observed by Carrapisco et al. [7] in their study of dry-cured sausages. Carrapisco et al. [7] also observed a prevalence of mesophiles and lactic acid bacteria (LAB), and although in our study mesophiles are indeed prevalent, psychrophiles are more prevalent than LAB. In fact, our LAB counts are quite low in the samples stored at room temperature, which was also described by Trejo et al. [10].

Although other studies showed similar results, it was still surprising that samples stored at 4 °C showed more microbial growth, since refrigeration is generally expected to reduce microbial growth. However, it is important to recall that the *Painho* samples stored at 4 °C also showcased higher values of a_w_ and pH (Table 3). The slightly higher a_w_ observed in refrigerated samples may have created conditions more favorable for microbial survival compared with samples stored at room temperature, where greater moisture loss may have occurred. 

Still, in general, values of the microbiological parameter, for both temperatures, were lower than those observed in other studies of dry-cured sausages [37,43,85], but closer to the ones described by Trejo et al. [10]. 

### 3.9. Sensory Analysis

Hardness was the only sensorial descriptor that showed significant differences for the interaction between ‘Storage time × Storage Temperature’, with values ranging between 47.8 ± 9.2 and 55.0 ± 7.6% (Table 9); no other significant differences were found. Panelists assigned higher hardness scores to samples stored at room temperature, aligning with the findings of Ramírez et al. [37] while the HHP treatment of their samples could have influenced those results. Ramírez et al. [37] also hypothesized that higher storage temperatures promote hardening—a change more easily perceived by panelists due to reduced moisture content. Similarly, our panel rated refrigerated samples higher in succulence and perceived room-temperature samples as saltier, which likely reflects the differences in moisture levels. This hypothesis is also supported by the lower levels of a_w_ found in room-temperature samples. It can also explain why panelists assigned higher values of salt perception to the room temperature samples, since a loss in moisture will affect the salt perception.

Regarding the color parameter, no strong correlation was found between the instrumental color parameters and the panelists’ scores. As Trejo et al. [10] suggest, this is likely because sensory analysis evaluates overall visual appearance, while instrumental color is limited to specific coordinate values.

Nonetheless, panelists in general showed a preference for the samples stored at 4 °C, giving those samples a higher score on the ‘Overall perception’, just as they did in parameters such as ‘Aroma intensity’, ‘Hardness’, and ’Succulence’. The ‘Off-flavor’ and ‘Off-aroma’ were scored quite low in general, which seems to go in line with the fact that lipid oxidation, which causes rancidity, was below the accepted levels.

## 4. Conclusions

Dry-cured products are highly valued for their sensory quality, making it fundamental to preserve those characteristics and ensure that there is no loss of quality during storage. While the vacuum packaging and the product’s intrinsic characteristics are effective at preserving many quality and safety attributes, they do not fully prevent slow oxidative and microbiological changes that can affect shelf life. Throughout this study, sliced vacuum-packaged *Painho* showed good oxidative stability (low TBARS), stable values of tocopherols, instrumental color remained as a whole unchanged during storage, and the samples remained uncontaminated of key pathogens over the 6-month storage period under both temperatures. Thus, based on the results obtained in this study, storage at room temperature showed slightly lower microbial counts and lower protein oxidation compared with refrigeration. These findings suggest that room-temperature storage could be a more suitable alternative for maintaining the physicochemical and microbiological stability of sliced and packaged *Painho de Porco Preto* during the 6-month storage period. 

This study, however, focused on a limited number of storage conditions and a specific type of packaging; therefore, future research should explore the influence of different packaging systems, different storage conditions, and additional physicochemical and sensory parameters to better understand the shelf life and quality evolution of traditional dry-cured sausages during storage.

## Figures and Tables

**Table 1:** Initial characterization of the *Painho* sausages.

	Protein (g/100 g)	Total Ash (g/100 g)	Moisture (g/100 g)	Dry Mater (g/100 g)	Chlorides (gNaCL/100 g)	Total Fat (g/100 g)
T0	24.2 ± 0.9	5.4 ± 0.7	30.9 ± 1.8	69.1 ± 1.8	3.4 ± 0.1	27.7 ± 6.1

Data are expressed as means ± SD.

**Table 2:** Effect of storage temperatures on the color coordinates L*, a*, and b* of *Painho* sausages.

Time	Temperature	Meat			Fat		
		L*	a*	b*	L*	a*	b*
T0	-	48.6 ± 3.2	31.1 ± 3.1	23.6 ± 2.2	64.9 ± 5.6	19.5 ± 5.2	21.8 ± 4.3 _a_
T2	Room temperature	47.6 ± 5.3	31.1 ± 3.5	23.5 ± 3.2	65.2 ± 4.3	19.9 ± 4.2	25.9 ± 2.7 _bc_
	4 °C	49.5 ± 2.3	30.9 ± 2.0	24.2 ± 1.5	63.5 ± 4.0	21.2 ± 3.5	23.9 ± 2.3 _ab_
T3	Room temperature	47.4 ± 2.4	32.0 ± 1.8	24.8 ± 1.5	66.1 ± 4.2	19.1 ± 3.4	25.5 ± 2.7 _abc_
	4 °C	48.4 ± 3.1	31.0 ± 3.0	23.5 ± 1.9	66.0 ± 5.8	18.2 ± 4.8	23.5 ± 3.2 _ab_
T4	Room temperature	48.0 ± 5.2	30.4 ± 5.4	24.5 ± 3.5	67.8 ± 4.4	16.8 ± 3.4	24.9 ± 2.8 _abc_
	4 °C	50.3 ± 5.9	28.0 ± 7.7	22.5 ± 3.6	64.9 ± 3.7	18.6 ± 3.8	24.2 ± 2.5 _ab_
T6	Room temperature	45.2 ± 3.1	29.4 ± 2.0	24.4 ± 3.0	65.2 ± 1.1	17.7 ± 2.4	28.3 ± 3.1 _c_
	4 °C	45.8 ± 2.7	30.7 ± 1.9	23.5 ± 2.0	65.3 ± 3.3	16.3 ± 2.7	23.3 ± 3.5 _ab_

Data are expressed as means ± SD. Distinct letters in the same column represent significantly different means (*p* < 0.05).

**Table 3:** Effect of storage temperature on pH and a_w_ of sausages.

Time	Temperature	pH	a_w_
T0	-	5.9 ± 0.0 _ab_	0.81 ± 0.0 _ab_
T2	Room temperature	5.7 ± 0.0 _cd_	0.80 ± 0.0 _b_
	4 °C	5.9 ± 0.0 _ad_	0.85 ± 0.0 _c_
T3	Room temperature	5.7 ± 0.1_acd_	0.83 ± 0.0 _ac_
	4 °C	5.9 ± 0.0 _ad_	0.86 ± 0.0 _c_
T4	Room temperature	5.7 ± 0.1 _cd_	0.84 ± 0.0 _c_
	4 °C	6.2 ± 0.5 _b_	0.85 ± 0.0 _c_
T6	Room temperature	5.6 ± 0.0 _c_	0.83 ± 0.0 _ac_
	4 °C	5.9 ± 0.0 _ad_	0.85 ± 0.0 _c_

Data are expressed as means ± SD. Distinct letters in the same column represent significantly different means (*p* < 0.05).

**Table 4:** Effect of storage temperature on antioxidant content of sausages.

Time	Temperature	α-Tocopherol (µg/g)	ɣ-Tocopherol (µg/g)
T0	-	7.0 ± 0.6	0.4 ± 0.0
T2	Room temperature	7.2 ± 0.8	0.4 ± 0.1
	4 °C	7.7 ± 0.7	0.4 ± 0.1
T3	Room temperature	7.3 ± 0.7	0.4 ± 0.1
	4 °C	7.1 ± 0.5	0.4 ± 0.1
T4	Room temperature	6.8 ± 0.5	0.3 ± 0.0
	4 °C	7.0 ± 0.8	0.4 ± 0.0
T6	Room temperature	7.0 ± 0.8	0.4 ± 0.1
	4 °C	6.8 ± 0.8	0.4 ± 0.0

Data are expressed as means ± SD.

**Table 5:** Effect of storage temperature on the lipid and protein oxidation of sausages.

Time	Temperature	Lipid Oxidation (mg MDA/kg Sample)	Protein Oxidation (nmol Carbonyls/mg Protein)
T0	-	1.0 ± 0.2	3.8 ± 1.0 _a_
T2	Room temperature	0.8 ± 0.1	4.6 ± 1.2 _a_
	4 °C	0.7 ± 0.1	4.4 ± 1.1 _a_
T3	Room temperature	1.0 ± 0.1	4.4 ± 1.0 _a_
	4 °C	1.1 ± 0.3	4.9 ± 1.6 _ab_
T4	Room temperature	0.8 ± 0.1	4.5 ± 1.4 _a_
	4 °C	0.9 ± 0.3	5.1 ± 1.3 _ab_
T6	Room temperature	0.9 ± 0.2	3.9 ± 1.6 _a_
	4 °C	0.9 ± 0.2	6.1 ± 1.2 _b_

Data are expressed as means ± SD. Distinct letters in the same column represent significantly different means (*p* < 0.05).

**Table 6:** Effect of storage temperature on the fatty acid profile (mg FAME/g of fat) of the *Painho* sausages.

Time	Temperature	Capric (10:0)	Lauric (12:0)	Miristic (14:0)	Palmitic (16:0)	Palmitoleic (16:1)	Margaric (17:0)	Margaroleic (17:1)	Stearic (18:0)	Oleic (18:1)	Linoleic (18:2)	Linolenic (18:3)	Arachidic (20:0)	Gadoleic (20:1)	Eicosadienoic (20:2)	Arachidonic (20:4)	Behenic (22:0)	Erucic (22:1)	Lignoceric (24:0)	SFA	MUFA	PUFA
T0	-	1.21 ± 0.91	0.41 ± 0.21	6.50 ± 1.80	97.34 ± 26.52	12.19 ± 3.50	0.77 ± 0.23	0.79 ± 0.25 _ab_	45.64 ± 13.29	129.46 ± 40.22	18.38 ± 5.56	1.29 ± 0.44	0.37 ± 0.15	2.21 ± 0.91	0.99 ± 0.32	0.09 ± 0.03	0.03 ± 0.02	0.25 ± 0.14	0.16 ± 0.07	152.43 ± 41.83	144.91 ± 44.67	20.75 ± 6.31
T2	Room temperature	0.61 ± 0.18	0.44 ± 0.12	5.32 ± 1.55	82.46 ± 24.37	11.94 ± 3.64	0.65 ± 0.18	0.65 ± 0.22 _a_	41.91 ± 12.56	113.82 ± 33.79	15.79 ± 5.01	1.00 ± 0.30	0.58 ± 0.19	2.54 ± 0.72	0.91 ± 0.25	0.63 ± 0.19	0.02 ± 0.02	0.11 ± 0.06	0.03 ± 0.05	132.02 ± 38.93	129.07 ± 38.23	18.32 ± 5.66
	4 °C	0.70 ± 0.36	0.49 ± 0.20	6.26 ± 3.07	98.61 ± 46.70	13.70 ± 6.60	0.83 ± 0.45	0.92 ± 0.46 _b_	49.00 ± 23.32	139.72 ± 65.94	20.91 ± 11.66	1.32 ± 0.67	0.66 ± 0.25	3.13 ± 1.34	1.15 ± 0.55	0.76 ± 0.36	0.03 ± 0.02	0.12 ± 0.04	0.02 ± 0.03	156.62 ± 74.30	157.58 ± 74.34	24.15 ± 13.23
T3	Room temperature	0.54 ± 0.11	0.42 ± 0.07	5.42 ± 0.69	88.54 ± 10.35	12.77 ± 1.54	0.68 ± 0.10	0.69 ± 0.10 _ab_	45.99 ± 5.62	125.46 ± 14.13	17.90 ± 2.88	1.07 ± 0.19	0.48 ± 0.07	2.57 ± 0.32	0.90 ± 0.13	0.53 ± 0.06	0.03 ± 0.01	0.07 ± 0.02	0.04 ± 0.01	142.16 ± 16.89	141.56 ± 15.97	20.40 ± 3.25
	4 °C	0.61 ± 0.14	0.46 ± 0.07	6.01 ± 0.85	95.80 ± 12.21	14.19 ± 1.91	0.72 ± 0.11	0.74 ± 0.11 _ab_	48.30 ± 5.77	137.78 ± 16.45	19.63 ± 2.52	1.17 ± 0.16	0.52 ± 0.06	2.86 ± 0.32	0.99 ± 0.09	0.63 ± 0.08	0.03 ± 0.01	0.08 ± 0.02	0.05 ± 0.01	152.48 ± 19.14	155.66 ± 18.70	22.41 ± 2.82
T4	Room temperature	0.53 ± 0.15	0.39 ± 0.08	5.17 ± 1.08	85.19 ± 17.28	12.28 ± 2.50	0.64 ± 0.15	0.64 ± 0.14 _a_	44.35 ± 9.44	121.69 ± 24.97	16.35 ± 4.32	0.93 ± 0.26	0.46 ± 0.11	2.39 ± 0.50	0.78 ± 0.17	0.45 ± 0.09	0.02 ± 0.01	0.07 ± 0.02	0.05 ± 0.01	136.79 ± 28.22	137.06 ± 28.08	18.51 ± 4.83
	4 °C	0.61 ± 0.19	0.43 ± 0.13	5.65 ± 1.69	90.21 ± 26.76	12.92 ± 3.81	0.70 ± 0.21	0.69 ± 0.20 _ab_	45.90 ± 13.61	128.30 ± 37.70	18.65 ± 5.68	1.07 ± 0.35	0.48 ± 0.14	2.55 ± 0.76	0.87 ± 0.26	0.50 ± 0.15	0.03 ± 0.01	0.07 ± 0.03	0.05 ± 0.02	144.05 ± 42.73	144.53 ± 42.47	21.10 ± 6.42
T6	Room temperature	0.62 ± 0.09	0.45 ± 0.04	5.70 ± 0.53	92.61 ± 8.77	13.02 ± 1.35	0.69 ± 0.08	0.67 ± 0.08 _ab_	47.66 ± 4.08	129.29 ± 13.61	17.76 ± 1.93	0.98 ± 0.12	0.48 ± 0.05	2.39 ± 0.31	0.76 ± 0.09	0.41 ± 0.06	0.02 ± 0.01	0.08 ± 0.02	0.04 ± 0.01	148.27 ± 13.47	145.44 ± 15.27	19.92 ± 2.17
	4 °C	0.54 ± 0.21	0.37 ± 0.17	4.82 ± 2.18	77.57 ± 35.00	11.02 ± 5.00	0.59 ± 0.26	0.57 ± 0.26 _a_	39.04 ± 17.79	108.35 ± 48.80	15.08 ± 6.99	0.84 ± 0.37	0.39 ± 0.18	1.98 ± 0.89	0.63 ± 0.28	0.36 ± 0.13	0.03 ± 0.01	0.05 ± 0.02	0.04 ± 0.01	123.37 ± 55.73	121.98 ± 54.94	16.91 ± 7.77

Data are expressed as means ± SD. Distinct letters in the same column represent significantly different means (*p* < 0.05).

**Table 7:** Effect of storage temperature on the nitrite and nitrate content of sausages.

Time	Temperature	Nitrate (mg/kg Sample)	Nitrite (mg/kg Sample)
T0	-	95.7 ± 5.9	0.2 ± 0.0 _a_
T2	Room temperature	83.3 ± 9.9	0.7 ± 0.1 _b_
	4 °C	94.5 ± 10.4	0.6 ± 0.3 _bc_
T3	Room temperature	49.9 ± 9.6	0.4 ± 0.2 _abc_
	4 °C	65.1 ± 11.4	1.2 ± 0.7 _d_
T4	Room temperature	43.8 ± 8.8	0.3 ± 0.1 _a_
	4 °C	56.8 ± 9.9	0.2 ± 0.0 _a_
T6	Room temperature	78.6 ± 7.7	0.6 ± 0.1 _bc_
	4 °C	91.5 ± 8.7	0.4 ± 0.1 _ac_

Data are expressed as means ± SD. Distinct letters in the same column represent significantly different means (*p* < 0.05).

**Table 8:** Effect of storage temperature on the microbiological parameters of sausages (log cfu g^−1^).

Time	Temperature	Mesophiles	Lab	Staphylococci	Yeast	Psychrophiles
T0	-	6.2 ± 0.6 _ab_	5.7 ± 2.3 _a_	2.3 ± 2.2 _a_	3.9 ± 0.7 _ab_	6.5 ± 0.7 _a_
T2	Room temperature	5.2 ± 0.5 _cd_	2.0 ± 2.5 _bcd_	5.1 ± 0.6 _b_	4.1 ± 0.5 _abc_	4.7 ± 0.2 _bc_
	4 °C	6.5 ± 0.6 _b_	5.8 ± 0.7 _a_	3.3 ± 2.5 _ab_	4.4 ± 0.7 _bcd_	6.0 ± 0.5 _ab_
T3	Room temperature	5.5 ± 0.5 _ac_	3.4 ± 2.5 _acd_	5.4 ± 0.4 _b_	4.0 ± 0.3 _abc_	4.7 ± 0.2 _bc_
	4 °C	6.1 ± 0.6 _abc_	3.8 ± 3.0 _ad_	4.1 ± 1.6 _ab_	4.2 ± 0.4 _a-d_	5.7 ± 0.4 _abc_
T4	Room temperature	4.5 ± 0.4 _d_	0.5 ± 1.5 _bc_	4.1 ± 1.5 _ab_	3.9 ± 0.3 _ab_	4.4 ± 0.3 _c_
	4 °C	6.2 ± 0.5 _ab_	5.5 ± 0.8 _a_	4.9 ± 0.4 _b_	4.9 ± 0.4 _d_	5.6 ± 0.4 _abc_
T6	Room temperature	4.6 ± 0.4 _d_	0.0 ± 0.0 _b_	2.1 ± 2.5 _a_	3.5 ± 0.3 _a_	2.0 ± 2.4 _d_
	4 °C	5.7 ± 0.7 _abc_	5.1 ± 0.9 _a_	4.9 ± 0.5 _b_	4.7 ± 0.4 _cd_	5.0 ± 1.9 _abc_

Data are expressed as means ± SD. Distinct letters in the same column represent significantly different means (*p* < 0.05).

**Table 9:** Effect of storage temperature on sensory analysis of sausages.

Time	Temperature	Color Intensity	Off-Color	Marble	Aroma Intensity	Off-Aroma	Hardness	Fibrousness	Succulence	Flavor Intensity	Off-Flavor	Salt Perception	Overall Perception
T0	-	71.2 ± 13.8	1.6 ± 4.3	67.4 ± 15.5	73.3 ± 14.0	0.6 ± 2.3	47.8 ± 9.2 _a_	31.2 ± 21.3	63.6 ± 17.0	69.5 ± 14.2	1.3 ± 3.7	53.4 ± 10.3	70.4 ± 14.2
T2	Room temperature	67.8 ± 15.9	1.4 ± 3.7	68.8 ± 15.5	66.5 ± 15.0	1.9 ± 3.6	51.4 ± 6.8 _ab_	30.6 ± 21.9	64.8 ± 13.6	69.7 ± 10.8	2.0 ± 4.2	53.5 ± 7.8	69.3 ± 12.5
	4 °C	70.4 ± 13.1	1.8 ± 3.6	67.8 ± 17.2	65.2 ± 15.8	1.2 ± 3.3	53.1 ± 6.6 _b_	32.0 ± 22.9	63.0 ± 14.5	66.5 ± 12.9	1.5 ± 3.5	53.4 ± 9.7	67.3 ± 13.0
T3	Room temperature	74.2 ± 10.8	0.9 ± 2.4	71.6 ± 12.7	68.0 ± 12.9	2.6 ± 4.8	51.6 ± 7.7 _ab_	32.9 ± 22.0	58.9 ± 15.8	67.8 ± 11.4	3.9 ± 7.3	54.1 ± 7.7	64.3 ± 13.8
	4 °C	77.0 ± 10.7	2.0 ± 3.1	70.5 ± 14.1	68.8 ± 12.7	2.6 ± 6.6	51.1 ± 8.4 _ab_	41.7 ± 62.8	60.9 ± 14.5	69.6 ± 9.9	2.6 ± 5.2	54.1 ± 7.6	67.3 ± 11.6
T4	Room temperature	72.3 ± 12.3	2.7 ± 4.4	66.5 ± 13.8	67.9 ± 14.0	4.2 ± 9.2	53.6 ± 7.1 _b_	28.6 ± 23.0	63.5 ± 13.5	67.0 ± 12.7	8.2 ± 16.4	58.2 ± 12.4	64.3 ± 16.3
	4 °C	71.1 ± 12.1	3.9 ± 4.8	68.3 ± 12.6	65.1 ± 12.4	4.9 ± 11.2	51.2 ± 10.1 _ab_	29.0 ± 24.6	67.0 ± 13.1	66.8 ± 14.1	5.7 ± 15.0	53.6 ± 9.4	66.2 ± 16.1
T6	Room temperature	76.2 ± 9.7	2.2 ± 4.1	72.5 ± 10.5	70.0 ± 9.6	3.1 ± 7.8	55.0 ± 7.6 _b_	29.8 ± 19.3	64.3 ± 13.2	70.5 ± 9.1	5.6 ± 10.5	54.6 ± 7.7	67.1 ± 16.8
	4 °C	74.3 ± 9.8	2.5 ± 3.8	70.7 ± 11.2	68.7 ± 11.2	2.5 ± 4.6	51.6 ± 6.7 _ab_	28.5 ± 20.2	66.9 ± 12.3	68.6 ± 9.4	3.6 ± 6.9	51.9 ± 5.5	67.9 ± 10.5

Data are expressed as means ± SD. Distinct letters in the same column represent significantly different means (*p* < 0.05).

## Data Availability

The original contributions presented in this study are included in the article. Further inquiries can be directed to the corresponding author.
